# More primary care patients regret health decisions if they experienced decisional conflict in the consultation: a secondary analysis of a multicenter descriptive study

**DOI:** 10.1186/s12875-016-0558-0

**Published:** 2016-11-10

**Authors:** Maria-Margarita Becerra-Perez, Matthew Menear, Stephane Turcotte, Michel Labrecque, France Légaré

**Affiliations:** 1 CHU de Québec Research Centre –Laval University, St-François d’Assise Hospital, 10 de l’Espinay, Quebec City, QC G1L 3L5 Canada; 2 Department of Family Medicine and Emergency Medicine, Faculty of Medicine, Laval University, 1050 avenue Ferdinard-Vandry, Quebec City, QC G1V 0A6 Canada

**Keywords:** Decision regret, Decisional conflict, Shared decision-making, Primary care, Dyads, Risk factors

## Abstract

**Background:**

We sought to estimate the extent of decision regret among primary care patients and identify risk factors associated with regret.

**Methods:**

Secondary analysis of an observational descriptive study conducted in two Canadian provinces. Unique patient-physician dyads were recruited from 17 primary care clinics and data on patient, physician and consultation characteristics were collected before, during and immediately after consultations, as well as two weeks post-consultation, when patients completed the Decision Regret Scale (DRS). We examined the DRS score distribution and performed ordinal logistic regression analysis to identify predictors of regret.

**Results:**

Among 258 unique patient-physicians dyads, mean ± standard deviation of decision regret scores was 11.7 ± 15.1 out of 100. Overall, 43 % of patients reported no regret, 45 % reported mild regret and 12 % reported moderate to strong regret. In multivariate analyses, higher decision regret was strongly associated with increased decisional conflict and less significantly associated with patient age and education, as well with male (vs. female) physicians and residents (vs. teachers).

**Conclusion:**

After consulting family physicians, most primary care patients experience little decision regret, but some experience more regret if there is decisional conflict. Strategies for reducing decisional conflict in primary care, such as shared decision-making with decision aids, seem warranted.

## Background

Over the past two decades, a growing international movement to increase patient involvement in healthcare decisions and development of various decision support interventions has taken shape [1–5]. However, many healthcare decisions are challenging [6], and negative experiences during or after decision-making can lead patients to have regrets about the choices that were made [7]. Evidence suggests that decision regret is a common phenomenon in healthcare and that it can reach high levels for some medical decisions [8]. Decision regret is associated with lower satisfaction with care, negative experiences with the healthcare system, and reduced quality of life [7, 9–11]. As such, it is increasingly viewed as an important patient-reported outcome measure as well as a proxy measure for the quality of healthcare decisions [12, 13].

Assessing decision regret is particularly important in the context of primary care. For many patients, the clinical encounter with their family physician is the first point of contact with the healthcare system. This is where they learn about health issues, have their problems assessed and diagnosed, and consider steps they can take to preserve or improve their health [14]. Primary care providers offer care across the lifespan, manage all but the most uncommon or unusual conditions, and ensure continuity and coordination of care provided at other levels of the healthcare system or by other professionals [14]. Primary care thus encompasses the widest possible spectrum of health conditions and is the forum where the greatest number and diversity of medical decisions take place [15, 16], making it a very relevant clinical context for the study of decision regret.

Yet surprisingly few studies have investigated decision regret in primary care. In a systematic review of 59 studies examining the extent and predictors of decision regret related to healthcare decisions, we identified only five studies conducted in family medicine practices [8]. Specifically, authors assessed the extent of decision regret related to decisions about hormone replacement therapy [17], cardiovascular disease prevention [18], use of antibiotics for acute respiratory infections [19, 20], and treatment choices for diabetes [21]. In three of these studies a low mean level of decision regret was observed among participants [18–20], but in two studies (on hormone replacement therapy and diabetes decisions) authors reported relatively high levels of regret [17, 21]. None of the five studies examined risk factors contributing to decision regret in their primary care patients, nor did they examine whether decision regret varies across the multiple types of healthcare decisions that take place in primary care. Therefore, we sought to estimate the extent of decision regret experienced by primary care patients and to examine the factors associated with regret.

## Methods

### Study design and source of data

We performed a secondary analysis of all data collected between January 2009 and April 2010 for the EXACKTE2 (EXploiting clinicAl Consultations as a Knowledge Transfer and Exchange environment) study [15, 22]. The EXACKTE2 study assessed how patients and physicians influence each other in consultations, based on a shared decision-making (SDM) model but in the absence of SDM interventions. The study was conducted in 17 primary care clinics in two Canadian provinces [15, 22]. French-speaking pairs of patients and physicians were recruited in a practice-based research network (PBRN) in the Province of Quebec consisting of twelve family practice teaching units with a total of about 250 family physicians, including residents, and over 300,000 consultations per year. English-speaking pairs were recruited at the Family Medicine Education and Research Network (FERN) of the Thames Valley Family Practice Research Unit, consisting of 29 family practices across Western Ontario, with about 200 family physicians and more than 100,000 consultations per year. These two PBRNs are networks of individual primary care offices offering both continuous care and walk-in consultations with family physicians.

### Data collection procedure

As the primary goal of the EXACKTE2 study was to explore the mutual influence of patients and physicians during consultations, authors of the study recruited one patient from each participating physician (i.e. unique patient-physician dyads only). After obtaining the physician’s consent, research assistants trained in data collection consecutively recruited patients in the waiting room who were interested in participating. A detailed description of the recruitment process is specified elsewhere [23]. Patients were eligible if they were ≥18 years old, able to read English or French, able to provide informed consent, not suffering from an acute condition requiring urgent medical attention (e.g. transfer to emergency department), and able to report on a decision they had made with their physician. After physicians and residents were presented with the study specifics and had agreed to participate, they provided written consent and sociodemographic data (e.g. age, sex, and year of licensure). After securing their consent, patients met their physicians. Consultations were audiotaped to assess patient involvement in decision-making. After the encounter, patients completed a questionnaire about a decision made during their consultation, their uncertainty about the decision, and their sociodemographic characteristics (e.g. age, sex, education, marital status). Two weeks later, research assistants phoned patients to collect data on their level of decision regret and quality of life.

### Measures

The measures included in this secondary analysis were selected from a systematic literature review conducted by the research team [8] and for the potential of their variables to predict decision regret as suggested by EXACKTE’s theoretical framework [15, 22].

#### Decision regret scale

The dependent variable for this study was decision regret, assessed using the Decision Regret Scale (DRS) [7]. The DRS consists of five statements: (1) It was the right decision; (2) I regret the choice that was made; (3) I would go for the same choice if I had to do it over again; (4) the choice did me a lot of harm, and (5) the decision was a wise one. Agreement with each statement is measured on a five-point Likert scale (1 = strongly agree to 5 = strongly disagree). Score of each item is converted to a 0–100 scale by subtracting 1 from each item and multiplying by 25. Scores from items 2 and 4 are reversed. To obtain a global score, all items are summed and the total is divided by 5. Scores range from 0 (no regret) to 100 (high regret), increasing by increments of 5. This instrument showed good reliability in our data (Cronbach’s alpha = 0.74).

#### Decisional conflict scale

Decisional conflict refers to a patient’s personal uncertainty about the course of action to take when the choices involve risk, loss, regret, or a challenge to their personal life values [24]. The Decisional Conflict Scale (DCS) captures uncertainty in decision-making and factors contributing to uncertainty such as feeling uninformed, being unclear about personal values, and feeling unsupported. The DCS consists of 16 items, each of which is measured on a five-point Likert scale (1 = strongly agree to 5 = strongly disagree). The scores are converted to a 0–100 scale by subtracting 1 from each item and multiplying by 25. To obtain a global score, all items are summed and divided by 16. Scores range from 0 (no decisional conflict) to 100 (high decisional conflict) [25] incrementing by a value of 1.56. The DCS showed good reliability (Cronbach’s alpha = 0.73 in this study).

#### SF-12 quality of life scale

Patients’ quality of life was assessed using the Short Form-12 (SF-12) [26]. This valid and reliable measure uses 12 items to measure functional health and well-being from the patient’s point of view. The SF-12 scores were calculated as documented in the manual provided by developers using an international standardized algorithm [27]. Briefly, the questions were combined, scored, and weighted to create easily interpretable scales for mental and physical health as perceived by the patient. Physical and Mental Health Composite Scores (PCS & MCS) were computed using the scores of 12 questions and range from 0 to 100, where a zero score indicates the lowest level of health measured by the scales and 100 indicates the highest level of health [27].

#### OPTION - third observer scale

The OPTION (Observing patient involvement) scale has good internal reliability (Cronbach’s alpha = 0.75 in this study) that assesses 12 SDM-related behaviors that clinicians can adopt to promote patient participation in decision-making during consultations [28]. Based on verbatim transcripts of all clinical encounters, two trained coders independently rated the audiotaped encounters using OPTION as third-party observers. The items were coded on a five-point scale (0 = non-performance of a behavior to 4 = performance of a behavior at high competency) [28]. An overall score is obtained by adding the scores of each item, dividing by 12, and multiplying by 25. Scores range from 0 to 100 increasing by increments of 2.08, with a high score indicating that the clinician practised numerous behaviors associated with SDM [28]. The inter-rater agreement between our four coders using an intraclass correlation varied between 0.68 and 0.95, indicating a substantial agreement between coders.

### Data analysis

We used descriptive statistics to summarize participant characteristics and examined scores and distributions on all scales. After excluding dyads for which at least one of the questionnaires was missing, participant characteristics were similar to those of the original study population. We imputed missing data for the DCS using its average score. We observed that the distribution of DRS scores did not meet normality assumptions according to a Shapiro-Wilk test and displayed a very high kurtosis index. After we verified that normalization of the DRS variable could not be achieved through logarithmic transformations, we recoded decision regret as a multinomial variable: “no regret” (DRS score = 0), “mild regret” (DRS score = 5–25) and “moderate to strong regret” (DRS score ≥30). As discussed in our systematic review [8], there is currently no consensus on appropriate cut-off points for the DRS and no guidance available to indicate what scores are clinically significant. However, our approach to categorizing decision regret is consistent with several other studies [10, 29–33] and offers a logical clinical interpretation of scores given that participants will score at least 30 if they disagree or respond “neither agree nor disagree” on any one of the DRS statements while responding only “agree” on all others.

Distributions on the DCS, SF-12, and OPTION scores were highly skewed toward lower decisional conflict, moderate-to-strong quality of life and fewer SDM behaviors, respectively. As they did not respect the linearity of the regression coefficients, we created categories based on the literature for DCS (clinically significant decisional conflict defined as a score ≥25/100 on the DCS [34–36]) and tested median/terciles/quartiles to achieve the most efficient maximum likelihood estimates for SF-12 and OPTION scales. We performed bivariate analyses using non-parametric tests (Fisher exact test or Chi square, if applicable, for categorical variables and Kruskal-Wallis for ordinal variables) to examine relationships between decision regret scores and 1) patient characteristics, including socio-demographics, decisional conflict, and quality of life scores, 2) physician socio-demographic characteristics, and 3) consultation characteristics, including type of decision (diagnostic, therapeutic or follow-up), length of consultation, and OPTION-3rd observer scores. Variable selection was based on our systematic review [8] and the potential for variables to predict decision regret. As a negligible cluster effect was associated with the province and clinics (there was no cluster effect at the physician level as there was only one patient per physician), we performed ordinal logistic regression analyses to evaluate the adjusted associations between decision regret and predictor variables. First, we added all variables into the regression model and then we conducted a backward elimination. Forward and bidirectional stepwise regressions were also conducted to verify results. All variables that were statistically significant in any of the three models were retained for the final model. The threshold p-value for all statistical analyses was 0.05. Statistical analyses were performed with SAS version 9.4 (SAS Institute Inc., Cary, NC, USA).

## Results

### Participant characteristics

Of the 382 eligible physicians who were approached, 274 (72 %) agreed to participate. Of the 430 eligible patients approached, 276 (64 %) agreed to participate. We collected 264 questionnaires from the physicians and 269 from the patients. A total of 258 unique dyads were analyzed. Table 1 details the characteristics of the patients, physicians, and consultations. Both patients and physicians were more likely to be females. The mean age for patients was 50 years old whereas the mean age for physicians was 38 years old. Patients were most often married or in a common-law relationship and approximately two-thirds had either a college or university education. A higher proportion of participating physicians were residents in family medicine. Consultations lasted on average 31 min and covered a range of decisions, most often related to follow-up care. Overall, patients reported relatively low decisional conflict and good quality of life, while raters using the OPTION scale observed low performance of SDM behaviors by physicians.

**Table 1:** Characteristics of patients, physicians and consultations

Characteristics	*n* = 258^a^
Patients	
Female, *n* (%)	179 (69)
Age, yr, mean ± SD	50.0 ± 17.8
Marital status, *n* (%)	
Married/Common law	162 (63)
Single/Separated/Widowed	96 (37)
Education, *n* (%)	
Secondary or none	93 (36)
College/Professional degree	81 (32)
University degree	81 (32)
DCS score	
Mean ± SD	12.1 ± 11.2
Median (IQR)	10.9 (0–21.9)
Score >25/100, *n* (%)	50 (20)
Physical health	
Mean ± SD	44.8 ± 11.9
Median (IQR)	47.5 (36.7–53.8)
Mental health	
Mean ± SD	49.8 ± 10.9
Median (IQR)	51.6 (43.5–57.9)
Physicians	
Female, *n* (%)	163 (63)
Age, yr, mean ± SD	37.9 ± 10.7
Professional status, *n* (%)	
Residents	144 (56)
Teachers	114 (44)
Consultation	
Type of decision, *n* (%)	
Follow-up	115 (45)
Diagnostic	85 (33)
Treatment	57 (22)
Length, min, mean ± SD	31.0 ± 16.0
OPTION-3^rd^ observer score	
Mean ± SD	24.7 ± 8.7
Median (IQR)	25.0 (18.8–29.2)

Note: *n* number of participants, *SD* Standard deviation, *IQR* Interquartile range

*DCS* Decisional conflict scale (score range 0–100, with higher scores indicating increased conflict)

Physical and Mental Health Composite Scores (PCS & MCS) of quality of life scale (score range 0–100, with higher scores indicating greater health)

OPTION-3^rd^ observer = Observing patient involvement (score range 0–100, with higher scores indicating greater shared decision-making behaviors evaluated by a 3^rd^ observer)

^a^Missing data: 4 and 3 for patients’ age and education respectively, 2 for physicians’ age, 1 for nature of decision and 7 for DCS

### Frequency of decision regret

**Fig. 1:**
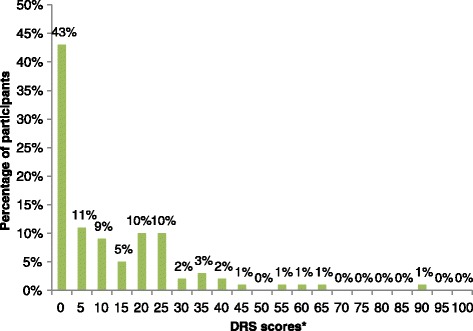
Frequency of patients’ decision regret scores. *DRS = Decision regret scale (score range 0–100, with higher scores indicating greater regret)

The global mean ± standard deviation (SD) decision regret score of patients was 11.7 ± 15.1 (median 5.0, IQR 0–20); 43 % of patients reported no decision regret (DRS score 0), 45 % reported mild regret (DRS score 5–25) and 12 % reported moderate to strong regret (DRS score 30 or higher). Figure 1 shows the distribution of decision regret scores. The distribution was skewed toward lower regret. Similar distributions and mean scores (10.4 to 13.6) were observed across the five DRS items. In general, the trend was towards low regret. Participants experiencing highest scores of regret showed similar characteristics to the whole sample in terms of patient and physician age and gender and length of consultation, but not in terms of decision type (more treatments), marital status (more were separated, single or widowed), education (lowest educated) and physician status (more residents). However, these differences did not reach statistical significance.

Participants experiencing highest scores of regret mentioned the following decision types most frequently: referral to a surgeon, spinal decompression treatment, starting or maintaining medication and waiting for referral to specialist. Participants experiencing lowest scores of regret mentioned the following decision types most frequently: pregnancy care, follow-up for several chronic conditions (e.g. diabetes and hypertension), annual exams and gynecological exams.

### Factors associated with decision regret

In bivariate analyses (Table 2), decision regret was associated with clinically significant decisional conflict (no regret 10.2 %, mild regret 24.4 % and moderate to strong regret 39.3 %, *p* < 0.0007). In this analysis, none of the other factors (patient and physician gender and age, patients’ marital status, education level and quality of life [PCS & MCS], physician status [resident or teacher], type of decision, length of consultation and OPTION-3rd observer scores) were significantly associated with higher decision regret. Although we did observe a slight association between decision regret and physician gender, namely higher regret when the physician was male, it was not significant. The higher intensity of regret among patients who consulted a male doctor affected the distributions in the “no regret” and “mild regret” categories, but not in the “moderate-to-strong regret” category (Table 2).

**Table 2:** Decision regret score according to patient, physician and consultation characteristics

	Decision Regret Score				
Characteristics	*n* ^a^	0 *n* (%)	5–25 *n* (%)	≥30 *n* (%)	*P*-value
Patients					
*Gender*					
Men	79	28 (35)	41 (52)	10 (13)	0.23^b^
Women	179	84 (47)	76 (42)	19 (11)	
*Age (years)*					
Mean ± SD	254	48.1 ± 16.2	50.5 ± 17.8	55.0 ± 22.1	0.21^c^
Median		48.1	50.3	59.2	
Age <40	87	41 (47)	37 (43)	9 (10)	0.16^b^
40–60	83	40 (48)	37 (45)	6 (7)	
Age >60	84	28 (33)	42 (50)	14 (17)	
*Marital status*					
Single/Separated/Widowed	96	38 (39)	42 (44)	16 (17)	0.10^b^
Married/Common law	162	74 (46)	75 (46)	13 (8)	
*Education*					
Secondary/none	93	32 (34)	47 (51)	14 (15)	0.11^b^
College/professional	81	35 (43)	37 (46)	9 (11)	
University	81	44 (55)	31 (38)	6 (7)	
*Decisional conflict* ^d^					
Mean ± SD	251	0 ± 0	14.9 ± 7.5	43.8 ± 13.9	0.0001^c^
Median		0	15	40	
DCS <25	201	97 (48)	87 (43)	17 (9)	0.0007^b^
DCS ≥25	50	11 (22)	28 (56)	11 (22)	
*Physical health* ^e^					
Mean ± SD	258	45.6 ± 11.8	44.5 ± 12.4	42.8 ± 10.7	0.38^c^
Median		49.2	47.7	44.0	
Score ≤ 36	62	23 (37)	31 (50)	8 (13)	0.49^b^
36< score ≤48	69	31 (45)	28 (41)	10 (14)	
48< score ≤54	63	28 (44)	27 (43)	8 (13)	
Score >54	64	30 (47)	31 (48)	3 (5)	
*Mental health* ^e^					
Mean ± SD	258	51.1 ± 11.0	48.8 ± 11.0	49.3 ± 10.0	0.07^c^
Median		53.9	49.9	52.6	
Score ≤ 43	63	22 (35)	34 (54)	7 (11)	0.28^b^
43< score ≤51	67	26 (39)	34 (51)	7 (10)	
51< score ≤58	65	30 (46)	25 (39)	10 (15)	
Score >58	63	34 (54)	24 (38)	5 (8)	
Physicians					
*Gender*					
Men	95	33 (35)	51 (54)	11 (11)	0.09^b^
Women	163	79 (48)	66 (41)	18 (11)	
*Age (years)*					
Mean ± SD	256	37.8 ± 11.3	38.1 ± 10.4	37.6 ± 9.8	0.85^c^
Median		34.3	35.2	38.2	
Age <31	89	39 (44)	41 (46)	9 (10)	0.98^b^
31–41	80	33 (41)	37 (46)	10 (13)	
Age ≥41	87	38 (44)	39 (45)	10 (11)	
*Professional status*					
Residents	144	58 (40)	65 (45)	21 (15)	0.14^b^
Teachers	114	54 (47)	52 (46)	8 (7)	
Consultation					
*Type of decision*					
Treatment	85	41 (48)	35 (41)	9 (11)	0.80^b^
Diagnostic	57	23 (40)	26 (46)	8 (14)	
Follow-up	115	48 (42)	55 (48)	12 (10)	
*Length (min)*					
Mean ± SD	258	31.2 ± 16.2	29.4 ± 15.6	30.0 ± 17.4	0.56^c^
Median		29.4	27.0	28.2	
≤21 min	84	33 (39)	43 (51)	8 (10)	0.33^b^
21–34 min	87	37 (43)	36 (41)	14 (16)	
>34 min	87	42 (48)	38 (44)	7 (8)	
*OPTION-3* ^*rd*^ *observer*					
Mean ± SD	258	24.6 ± 8.3	24.8 ± 8.5	25.0 ± 10.8	0.98^c^
Median		24.4	25.0	25.0	
Score ≤25	128	57 (45)	57 (45)	14 (10)	0.94^b^
Score >25	130	55 (42)	60 (46)	15 (12)	

*n* Number of participants

*SD* Standard deviation

^a^Missing data: 4 and 3 for patients’ age and education, respectively; 2 for physicians’ age, 1 for nature of decision and 7 for DCS

^b^Chi-square or Fisher exact test

^c^Kruskal-Wallis test

^d^The decisional conflict score (DCS) was dichotomised to the clinically significant cut-off (see Methods)

^*e*^Physical and Mental Health Composite Scores (PCS & MCS) of quality of life scale (score range 0–100, with higher scores indicating greater health)

In multivariate analyses, the factors that emerged as significantly associated with higher decision regret for patients in the ordinal logistic regressions were higher patient age, lower patient level of education, decisional conflict, male (vs. female) physicians, and resident status (vs. teachers) (Table 3). In the final adjusted model, the same variables remained significantly associated with decision regret, with the strongest predictor being decisional conflict. The ordinal logistic regression model respected the proportional odds assumption (*p* = 0.57), meaning that the OR between “no regret” vs “mild”/“moderate-to-strong” regret and “no regret”/“mild” vs “moderate-to-strong” regret were assumed to be the same. We also found a cluster effect associated with the province (0.03) and the clinics (0.02), although not statistically significant. In the present study, this effect had no impact on the final results.

**Table 3:** Multivariate analyses of decision regret – Ordinal logistic regression models

Characteristics	Full model			Final model^a^		
	OR	95 % CI	*p-value*	OR	95 % CI	*p-value*
Patients						
*Gender*						
Men	0.99	0.54–1.80	0.96			
Women	1.00					
*Age*						
<40 years	0.58	0.29–1.15	0.12	0.63	0.34–1.18	0.15
40–60	0.42	0.21–0.84	0.01	0.47	0.25–0.88	0.02
>60 years	1.00			1.00		
*Marital status*						
Alone	0.94	0.54–1.67	0.84			
In couple	1.00					
*Education*						
Secondary or none	1.52	0.77–3.00	0.23	1.96	1.06–3.66	0.03
College/Professional degree	1.77	0.91–3.45	0.09	1.86	0.98–3.54	0.06
University degree	1.00			1.00		
*Decisional conflict*						
Score ≤25	1.00			1.00		
Score >25	3.22	1.60–6.46	0.001	3.42	1.79–6.52	0.0002
*Physical health*						
Score ≤36	1.51	0.69–3.29	0.30			
36< score ≤48	1.43	0.68–3.02	0.34			
48< score ≤54	1.15	0.55–2.41	0.71			
Score >54	1.00					
*Mental health*						
Score ≤43	2.11	0.96–4.67	0.06			
43< score ≤51	2.08	0.98–4.40	0.05			
51< score ≤58	1.68	0.78–3.62	0.18			
Score >58	1.00					
Physicians						
*Gender*						
Men	1.78	1.00–3.20	0.05	1.78	1.05–3.01	0.03
Women	1.00			1.00		
*Age*						
<31 years	0.69	0.28–1.67	0.41			
31–41 years	0.96	0.47–1.97	0.91			
≥41 years	1.00					
*Professional status*						
Residents	2.56	1.22–5.49	0.01	1.72	1.02–2.90	0.04
Teachers	1.00			1.00		
Consultation						
*Type of decision*						
Treatment	0.74	0.40–1.37	0.34			
Diagnostic	1.27	0.64–2.55	0.49			
Follow-up	1.00					
*Length*						
Duration ≤21 min	1.39	0.70–2.79	0.35			
21< duration ≤34 min	1.36	0.70–2.64	0.37			
Duration >34 min	1.00					
*OPTION- 3* ^*rd*^ *observer*						
Score ≤25	1.03	0.60–1.75	0.93			
Score >25	1.00					

^a^Variables that were significant in any of the three stepwise regressions (backward, forward, stepwise) were retained for the final adjusted model

## Discussion

Our study results indicate that the overall extent of decision regret experienced by primary care patients following consultations with family physicians or residents in family medicine is low. To the best of our knowledge, this study is the first to examine factors associated with decision regret in primary care settings. We observed a significant association between patient decision regret and patient decisional conflict in bivariate analyses. In multivariate analyses, we found that higher decision regret was strongly associated with clinically significant decisional conflict, and weakly associated with male physicians and their status as residents, while regret was lower among patients between the ages of 40 and 60 years and those with a university education. Our results lead us to make three main observations.

First, the extent of decision regret found in this study is consistent with the literature. Our systematic review identified 44 studies reporting mean scores on the DRS, leading to an overall unweighted mean ± SD score of 16.5 ± 10.9 out of 100 across all studies [8]. Furthermore, 14 studies reported the proportion of patients who experienced no regret (DRS score = 0), which ranged from 14 to 98 % across studies, with an unweighted mean ± SD of 59.0 % ± 24.6 %. Our results suggest that primary care patients experience similarly low levels of decision regret as do patients in other clinical contexts. A difficulty that remains, however, is how to interpret these results. Several authors have characterized DRS scores ranging from 5 to 25 on 100 as mild decision regret [10, 29–33, 37], yet no consensus exists and the clinical significance of different scores and categorizations remains to be determined. While it is reassuring that most patients report relatively low levels of decision regret in primary care settings where the bulk of healthcare services are delivered, further research is needed to identify valid and reliable cut-off points for distinguishing clinically significant regret and its impact on patient health.

Second, our results are similarly consistent with the literature regarding the influence of decisional conflict on decision regret. A recent meta-analysis of ten clinical trials found a strong positive association between decisional conflict and decision regret (OR = 5.52; 95 % CI 3.35–9.12) [36, 38] and our own systematic review has shown that decisional conflict was among the predictors most frequently and significantly associated with decision regret [8]. Decisional conflict typically occurs when patients face difficult decisions for which multiple reasonable options exist, as well as when patients feel unsupported in the decision-making process. Decisions that are made in a context of uncertainty can lead to decision regret, especially when there is no clearly preferable clinical option. Several studies have shown that regret is also a common consequence of preference-sensitive decisions [9, 39]. Our results thus justify efforts to identify and reduce patients’ decisional conflict in primary care consultations.

One ideal measure for identifying decisional conflict in clinical settings is the SURE (Sure of myself; Understanding information; Risk-benefit ratio; Encouragement) tool [40]. SURE is a short, four-item clinically oriented screening tool for decisional conflict that has been validated in primary care settings [34]. Identifying patients experiencing uncertainty about the choices they face and the consequences of these choices may prompt physicians to increase supports for decision-making. These supports may take the form of SDM facilitated by decision aids. High-quality evidence suggests that decision aids reduce patients’ decisional conflict and also increase decision-specific knowledge and overall satisfaction with care [41]. Yet, despite the evidence for their benefits, decision aids and SDM generally are not widely implemented in clinical practice [42, 43]. Broader adoption of SDM strategies to address factors contributing to decisional conflict [44], such as feeling uninformed about options, feeling uncertain about risks and benefits of options, or feeling unsure about values and preferences related to choices or consequences, should help reduce the decision regret that some patients experience [45].

Third, some of our findings differ from those of previous studies. For instance, our multivariate analyses revealed that patient age and education were associated with decision regret. Yet our systematic review, albeit mostly of studies conducted mostly in non-primary care settings, indicated that these factors rarely predict regret [8]. We observed higher regret in older patients, which may be a consequence of higher rates of adverse outcomes in these patients or to their preferences for more paternalistic decision-making approaches with physicians [46]. Higher regret was also observed in patients with lower education levels, possibly due to lower rates of health literacy in these populations [47, 48]. Low health literacy can limit patients’ capacity to understand basic health information and acts as a barrier to their participation in healthcare decisions [47, 48]. Interestingly, our study also found that regret was higher when physicians were male and were residents. Why patients of male physicians had higher regret is unclear, as a recent systematic review indicated no clear impact of physician gender on a number of patient-doctor communication characteristics [49]. But other studies have suggested that female physicians adopt more of a partnership style and a more patient-centered approach than their male colleagues [50–52]. As there is no consensus on the level of decision regret that is clinically significant and the association between physician gender and regret did not affect the highest scores, it is likely that this statistical association was not clinically significant. Residents, however, have been shown to lack some communication and SDM skills [43], which may explain why their patients had higher decision regret scores. These latter results should be interpreted cautiously, however, as the strength of association with decision regret was variable and associations had confidence intervals with values close to 1. They cannot be interpreted with the same confidence as our findings regarding decisional conflict.

This study has some limitations. First, as this was a secondary analysis of an observational descriptive study we were not able to explore the effect of time on decision regret scores. It could be that levels of decision regret were underestimated because of the short time period (two weeks) between the initial consultation and the assessment of decision regret. Patients may not have had sufficient time to develop feelings of regret about their decisions or experience adverse outcomes. Unfortunately we cannot compare this with the literature because the intervals between decision and regret most frequently used have been one, three, six and 12 months [8]. Longitudinal and prospective research is needed to examine the emergence of regret and its subsequent trajectory to explore the possible conceptual division of regret into two categories, immediate and delayed [13]. A potential desirability bias may also have contributed to low regret scores, particularly related to the phone call follow-up questionnaires. Patients may not have wanted to compromise their relationships with their physicians by reporting high levels of regret about their decisions. This bias was not evaluated in the context of decision regret but one study has shown that mail administration of SF-12 scored lower total averages than telephone administration [53]. In addition, the study was conducted on a convenience sample, so a selection bias is possible especially as we had no data about those individuals who refused to participate in the study and because recruited physicians were members of practice based networks and not randomly selected. As the current state of knowledge does not permit accurate interpretation of the DRS scores regarding the clinical impact of decision regret on physical and mental health, future research is needed to examine this impact and the types of decisions that lead to more regret in patients. Also, we may have overinterpreted our findings due to lack of sufficient information. For example, the difference in decision regret between patients with secondary and lower levels of education and a University education was 8 %, yet its significance was only *p* = 0.11. Future research is needed with larger samples to re-evaluate our interpretation. Finally, although we found no impact of the cluster effect on the final results, this information can be used now to calculate a sample size for a future cluster trial in Canadian primary care settings with decision regret as the primary outcome.

## Conclusion

Most primary care patients experience relatively low levels of decision regret after their consultations with family physicians, although higher decision regret was found when patients experienced higher decisional conflict. Decisional conflict during consultations can be addressed with effective decision support interventions aiming to foster SDM, such as patient decision aids. Further research is needed to explicitly examine the trajectory of decision regret over time in primary care settings and to identify its clinically significant effects.

## Abbreviations

CI: Confidence interval
DCS: Decisional conflict scale
DRS: Decision regret scale
OPTION: Observing patient involvement
OR: Odds ratio
SD: Standard deviation
SDM: Shared decision-making
SF-12: Short Form (12 items) of Quality of Life Survey
